# A study of macro-, meso- and micro-barriers and enablers affecting extended scopes of practice: the case of rural nurse practitioners in Australia

**DOI:** 10.1186/s12912-019-0337-z

**Published:** 2019-04-02

**Authors:** Tony Smith, Karen McNeil, Rebecca Mitchell, Brendan Boyle, Nola Ries

**Affiliations:** 1 Department of Rural Health, The University of Newcastle, 69A High Street, Taree, NSW 2430 Australia; 2 Faculty of Health and Medicine, University of Newcastle, Callaghan, Australia; 3 Faculty of Business and Law, University of Newcastle, Callaghan, Australia; 4 Faculty of Law, University of Technology Sydney, Ultimo, Australia

**Keywords:** Advanced practice, Rural and remote, Interprofessional teamwork, Role extension, Institutional models, Health care management

## Abstract

**Background:**

Shortages of skills needed to deliver optimal health care in rural and remote locations raises questions about using extended scopes of practice or advanced practice models in a range of health professions. The nurse practitioner (NP) model was introduced to address health service gaps; however, its sustainability has been questioned, while other extended scope of practice roles have not progressed in Australia. This study aimed to explore the experiences and perceptions of NPs and their colleagues about barriers to and enablers of extended scope of practice and consider the relevance of the findings to other health professions.

**Methods:**

Semi-structured, in-depth interviews were conducted with primary, nurse practitioner informants, who were also invited to nominate up to two colleagues, as secondary informants. Data analysis was guided by a multi-level, socio-institutional lens of macro-, meso- and micro-perspectives.

**Results:**

Fifteen primary informants and five colleagues were interviewed from various rural and remote locations. There was a fairly even distribution of informants across primary, aged, chronic and emergency or critical care roles. Key barriers and enablers at each level of analysis were identified. At the macro-level were legal, regulatory, and economic barriers and enablers, as well as job availability. The meso-level concerned local health service and community factors, such as attitudes and support from managers and patients. The micro-level relates to day-to-day practice. Role clarity was of considerable importance, along with embedded professional hierarchies and traditional role expectations influencing interactions with individual colleagues. Given a lack of understanding of NP scope of practice, NPs often had to expend effort promoting and advocating for their roles.

**Conclusions:**

For communities to benefit from extended scope of practice models of health service delivery, energy needs to be directed towards addressing legislative and regulatory barriers. To be successful, extended scope of practice roles must be promoted with managers and decision-makers, who may have limited understanding of the clinical importance. Support is also important from other members of the interprofessional health care team.

**Electronic supplementary material:**

The online version of this article (10.1186/s12912-019-0337-z) contains supplementary material, which is available to authorized users.

## Background

The shortage of health care practitioners in non-metropolitan areas is a significant concern globally, including in Australia [1–3]. There is an argument, however, that the shortage is a maldistribution of skills, rather than personnel, raising questions about development of innovative models of care, creating new health care roles and expanded or extended scopes of practice for existing health professions [4–7].

The nurse practitioner (NP) role is an extended scope of practice model with potential to increase the effectiveness of healthcare by addressing service gaps in health workforce capabilities [8–11]. In Australia, to become endorsed as a NP, a registered nurse (RN) must provide evidence of at least 5000 h of clinical experience at an advanced practice level, successfully complete an approved Master’s degree (or equivalent) and comply with the NP practice standards. The scope of the NP role extends beyond the traditional RN scope of practice, enabling NPs to conduct advanced health assessments and diagnoses, order and interpret diagnostic tests, prescribe some medications, and refer patients to other health care providers [12, 13]. Duffield et al. compared advanced practice nursing internationally, including NP roles, finding many similarities, though inconsistencies in nomenclature that need to be standardised [14]. It was considered that there was now national consistency between jurisdictions within Australia, with the NP role definition stating that autonomous, as well as collaborative practice is expected of NPs [15]. However, the capacity of NPs to provide definitive care across the entire care continuum is limited by lack of access to the Medicare Benefits Schedule (MBS) and Pharmaceutical Benefits Schedule (PBS) [13, 16]. Use of these publicly funded, Australian Government payment reimbursement schemes is accessible to private nurse practitioners who have a provider number but not to those working in the public sector [13], which includes public hospitals and public community or primary health care facilities.

The NP model was initially introduced as a potential way to address health service gaps in rural and remote areas [9, 10]. From a human resource management perspective, positive impacts of NPs have been noted, including improving workplace cultural, job satisfaction and retention [17]. Significantly, such positive impacts reportedly extended beyond the NPs themselves, the presence of the NPs also improving medical practitioners’ intentions to remain in rural practice [17].

However, the sustainability of the NP model has been questioned, with criticism that NPs have not made a marked difference to gaps in rural health services [18]. In Australia, NPs represent only 0.52% of the registered nursing and midwifery workforce [12]. Despite their potential, there is evidence that implementing such an important healthcare human resource reform has met with significant barriers [19] and NPs remain an underutilised resource [11]. It also seems that opportunities for other significant health workforce innovations and reform, including broadening scopes of practice of other health professions, have not progressed in Australia [5]. Health workforce reform recommendations have been made in substantial, publicly funded reports over several years [20–22]; yet, there remains a sense that more could be done [22]. It has been argued that significant productivity gains, including better access to care in rural and remote areas, would eventuate from changing practice and funding models [21, 23]. Recommended changes include adjusting practitioners’ skill mix and expanding or extending scopes of practice [21], among other health workforce reforms. The 2015 Productivity Commission research paper on efficiency in the Australian healthcare system highlighted potential benefits, including improved timeliness and access to care, increased job satisfaction for health workers and reduced costs of service delivery [22]. Meanwhile, in this context, little attention has been given to factors that have enabled the NP model to develop, though perhaps less so than it could, why its potential is not fully realised and how that experience might inform the development of extended scope of practice models in other health care disciplines [24–26].

### Study aims

The primary aim of this study was to explore the experiences and perceptions of NPs who work in non-metropolitan settings, as well as their colleagues (where possible), about the barriers to and enablers of extended scope of practice roles. Using the NP model as an example, the secondary aim was to use an established socio-institutional theoretical model of macro-, meso- and micro-perspectives [27–29] to reflect on how such barriers and enablers may be generalised to extended scope of practice roles in other health professions, especially in the context of rural and remote practice. The socio-institutional lens predicts a complex interaction between formal and informal factors in shaping individual and organisational behaviour and, ultimately, practice outcomes [19].

## Methods

The researchers were from varied backgrounds in management, organisational behaviour, law, and clinical and health service delivery and research, including rural health. They have an interest and previous research experience in collaborative team-based care and interprofessional boundary-work. The qualitative study design and methodology was approved by the University of Newcastle Human Research Ethics Committee. All participants were provided with a participant information statement prior to giving written informed consent to be interviewed under the approved conditions.

### Study design

The study design respects pre-existing theoretical development, which necessitated a design between the extremes on a methodological continuum from exploration to explanation. There is an extant insight from socio-institutional theory into expectations that professions implement change based on both formal, regulatory and institutional, as well as informal, normative factors at the boundaries between professions [19]. Thus, the preliminary theoretical model of macro-, meso- and micro-level factors was recognised from the outset, as referred to above. On the other hand, however, there is a paucity of pre-existing theory on how regulatory, institutional and normative factors affect the implementation of extended scope of practice roles, particularly in the rural context.

The unit of investigation in this study was the health professional working in an extended scope of practice role in a rural or remote setting. Each ‘case’ sheds light on the barriers and enablers of the role, thus producing data to address the research aim and objectives [30, 31]. However, the overall objective is to reflect more broadly on the role, not on individual cases, and to examine how individuals’ experiences of the nurse practitioner role might inform the development of extended scope of practice roles in rural areas more generally.

### Recruitment and data collection

Recruitment targeted rural and remote endorsed nurse practitioners working in extended scope of practice roles in the provision of health and social care as the primary informants. At the end of their interview, primary informants were asked if they would like to nominate up to two work colleagues who were likely to have knowledge of their role to be interviewed as secondary informants. While the initial interviews gave an ‘insider’ perspective, the intention of the interviews with the colleagues was to help validate the data and to increase the depth of understanding from the perspective of a ‘knowledgeable outsider’ or observer from the same ‘arena’ [32].

Primary informants were initially called on to volunteer via emailed invitations from the New South Wales (NSW) Nurses and Midwives Association, targeting nurse practitioners who worked in an extended scope of practice role in a public health facility in a regional, rural or remote location. Given the limited response to the initial recruitment, the Australian College of Nurse Practitioners was asked to distribute invitations to members in all Australian States and Territories. It was not obligatory for primary informants to nominate a colleague and most chose not to and were not required to explain why not, for ethical reasons.

Data collection used semi-structured, in-depth telephone and face-to-face interviews conducted by two researchers (KMc & BB), at times and locations convenient to prospective informants. Both interviewers were extensively experienced in qualitative research. Neither was a health professional, adding to the degree of objectivity and avoiding issues of assumed knowledge on the part of either the interviewer or interviewee. The schedule of interview questions, which were informed by deductive analysis of literature and extant knowledge of the topic, is included in the Additional file 1. While the type of questions was similar, interviews with the primary informants were of 30 to 90 min’ duration and yielded more depth compared with secondary informants’ interviews, which took between 20 and 60 min. All interviews were recorded and transcribed verbatim by a specialised transcription service with which the University has a confidentiality agreement. All informants were given the option of reviewing and editing their transcript, though only 11 chose to do so, 7 of who made changes.

### Data analysis

Data from primary and secondary informants were pooled for analysis, as they addressed the same study aim and similar interview questions. In accordance with Huberman et al.’s advice for analysing interviews across multiple ‘case’ contexts, data for individual informants was analysed separately and where common themes emerged they were combined [33], as presented in Table 2. Data analysis was undertaken from the perspective of the socio-institutional lens referred to above as preliminary theoretical framework, which considers formal and informal factors that shape behaviour across professional boundaries. While acknowledging pre-existing theoretical insights, maintenance of ‘theoretical detachment’ [34] allowed development new insights, as appropriate, based on the practitioners experiences.

Two of the researchers (KMc & TS) independently coded the data for content and meaning [35] using manual data analysis. Techniques of coding, indexing and labelling data were based on well-established methods [36]. Early inductive data analysis of transcripts and field notes paralleled later data collection. Deductive analysis was additionally informed by the literature. The cumulative process of pattern analysis and descriptive and interpretive coding focused on how the barriers and enablers informed the socio-institutional framework. Data saturation was identified as the point where continued data analysis and sampling provided no discernible new themes or linkages between themes [37].

## Results

A breakdown of the key characteristics of the 20 study informants is given in Table 1. They were all from locations categorised as MMM2 to MMM7 using the Modified Monash Model (MMM) classification [38]. All 15 primary informants worked in extended scope or advanced practice nursing roles; however, one was an endorsed NP but not employed as such, reflecting local availability of NP positions, and two were NP candidates but working in extended scope roles. There were five secondary informants nominated, one of who was an endorsed NP not working in an NP role at the time and another NP candidate not working in an extended scope of practice role.

**Table 1:** Characteristics of the nurse practitioner (NP) study informants and their colleagues

Descriptive Characteristics	Number
*Primary Informants*	15
Nurse Practitioners (1 not employed in an NP role)	13
NP Candidates (1 RIPERN* endorsed, 1 CNC †)	2
*Secondary Informants (Colleagues of the Primary Informants)*	5
Nurse Practitioner – not employed as an NP	1
Nurse Practitioner candidate	1
Allied Health Professionals	3
*Locations* ‡ *(including Colleagues)*	
MMM2	4
MMM3	6
MMM4	4
MMM5	3
MMM6	1
MMM7	2
*Specialty (including Colleagues)*	
Primary Care	6
Chronic Care (including 2 colleagues) §	5
Aged Care (including 2 colleagues) §	5
Emergency or Critical Care (including 1 endorsed NP colleague)	5

*Rural and Isolated Practice Endorsed Registered Nurse

† Clinical Nurse Consultant

‡ Modified Monash Model (MMM) Classification [38]

§ 1 NP informant worked across both aged and chronic care specialties

**Fig. 1:**
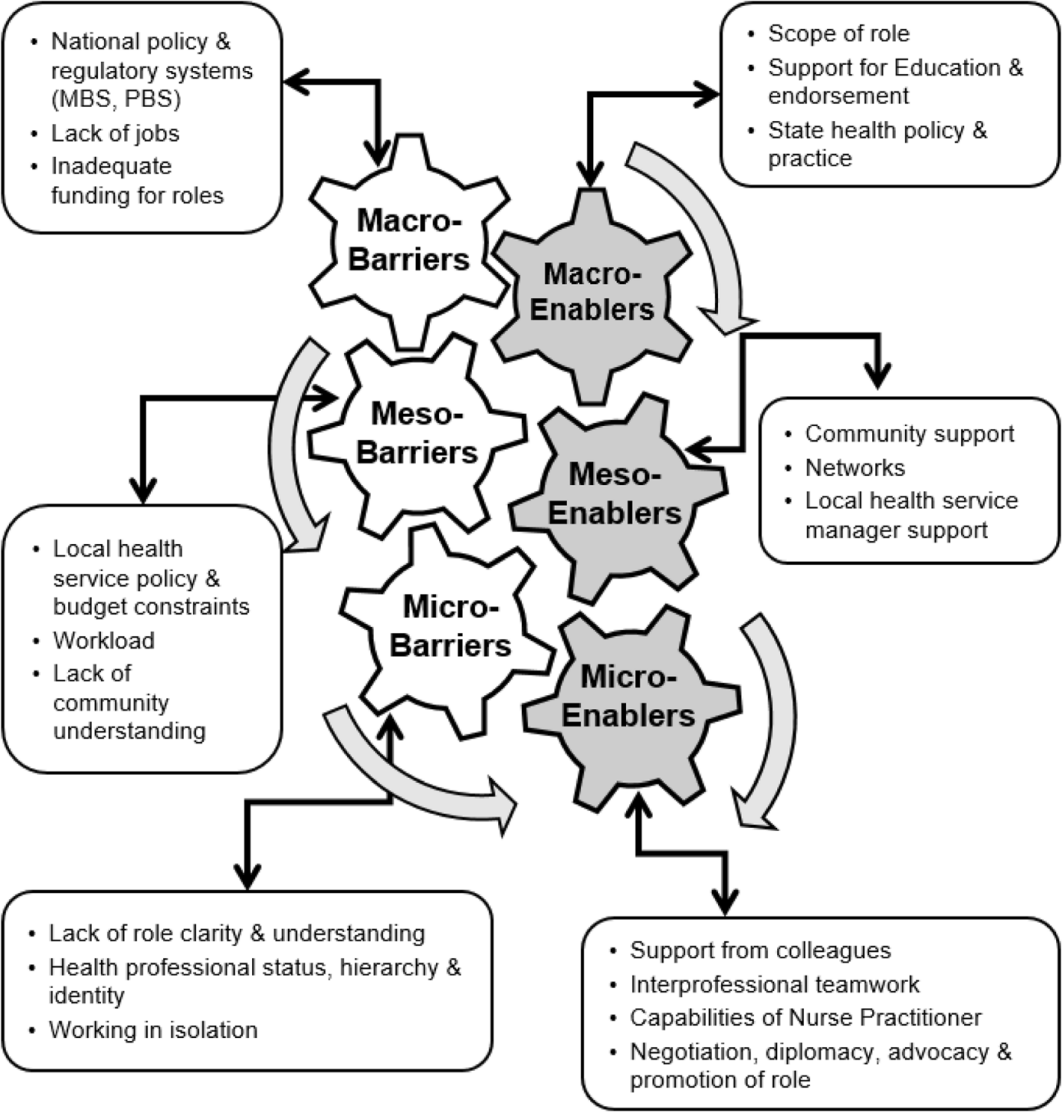
The barriers to and enablers of extended scope of practice in rural and remote Australia. (Adapted from Mulvale, Embrett and Razavi [20] and Nelson et al. [21])

Data categories relevant to both barriers and enablers are listed in Table 2, together with a definition of the macro-, meso- and micro-terminology, guided to varying degrees by similar definitions used elsewhere [13, 39–41]. Figure 1 is a diagrammatic representation of the emergent theoretical model, the overall structure of which was informed, in part, by the ‘gearing up’ model described by Mulvale, Embrett and Razavi [39]. The barriers and enablers are summarised below, with selected, representative and illustrative quotations from informants. NPs’ and colleagues’ quotations are numbered, with the latter indicated by the letter ‘C’ after the number.

**Table 2:** Definition of the macro-, meso- and micro-components and list of barriers and enablers evident in the model shown in Fig. 1

Barriers	Enablers
Macro - Perceived structural, legal, regulatory and economic external conditions that are beyond the influence of individual organisations or practitioners.	
• National policy and regulatory systems (MBS & PBS) • Lack of jobs • Inadequate funding of roles	• Scope of role • Support for education and endorsement • State health service policy and practice
Meso - Local institutional factors and influences, as well as community issues that often characterise or define the parameters of service delivery.	
• Local health service policy and budget constraints • Workload • Lack of community understanding	• Community support • Networks • Local Health Service Manager support
Micro - Day-to-day practice and attributes or characteristics of individual practitioners and their practice environments that affect how services are delivered.	
• Lack of role clarity and understanding • Health professional status, hierarch & identity • Working in isolation	• Support from colleagues • Interprofessional teamwork • Capabilities of Nurse Practitioner • Negotiation, advocacy, diplomacy & promotion of role

### Macro-barriers

At the macro-level were the perceived legal, regulatory, and economic barriers and enablers. Included were national policy and regulatory systems, such as the Medicare Benefits Schedule (MBS) and the Pharmaceutical Benefits Scheme (PBS). The NP scope of practice is constrained by regulations governing health care funding within Australia [13, 42, 43]. In 2010, NPs gained limited access to the MBS and PBS, enabling their patients to receive rebates for specific NP services and to access subsidies for some common medications [43]. Federal law limits the medications in the PBS that can be prescribed by NPs, however, permission to prescribe is dependent on State regulations. Meanwhile, NPs in the private health sector can apply for a Medicare provider number to bill for some consultations, request specified pathology and radiology services, or refer to medical specialists. Such anomalies and complexities contribute to an overly complicated practice environment, as evidenced elsewhere [13] and observed by study informants.

*Medicare, it's* [expletive]*, in a nutshell. It just makes our lives really difficult. We've got four … time-based item numbers. That's all we can bill for. …* [Consequently] *I probably only generate enough income to support a third of my role, a third of my salary.* (NP11)

There was a perception by several informants that the regulatory system was obstructive and not necessarily in the best interest of patient care.

*GPs* [General Practitioners] *are the current gatekeepers. Goals set by GPs are more medical goals, and not necessarily based on the needs of the patient (NP1).*

Consequently, given limitations on access to provider numbers, nurse practitioners find ways to work-around regulator barriers, creating inefficiencies.

*If I want to organise x-ray and pathology outside hospital have to get a GP to authorise. I don’t have a Medicare provider number as public health employee. Wastes a lot of time ringing GPs to review the patient and then get them to order x-rays or pathology. (NP7).*

There was also a perception that there were limited employment opportunities for endorsed NPs. Some informants had moved towns and in some cases from one State to another to secure an NP position. Others recounted situations where endorsed NPs could not get employment or could only find a part-time work. It was suggested that the problem was a lack of organisational support after endorsement.

*Often they think they've got these great models for* [NP] *candidates but it's what happens thereafter that seems to fall apart because we get all these people through we don't actually have jobs for them so they're endorsed but they're not working as nurse practitioners.* (NP14).

Lack of funding was also noted as a significant barrier. One informant, NP3C, expressed frustration that funding made available more than 12 months earlier in the local health district to employ NPs had not been used, concluding that the organisation *“does not like NPs”*, in spite the particular health district being “*… the place where Nurse Practitioners were born, where they were designed for”*. Similarly, NP4 saw potential for the role to address services gaps in rural health but *“for some reason, the funding is just not coming through. It’s being wasted on other things”*.

### Macro-enablers

In spite of organisational and institutional challenges to implementing NP roles, the role itself was viewed positively. High levels of education, accreditation, experience and responsibility afforded NPs higher levels of remuneration and autonomy (NP3, NP5, NP12). Informants gained prestige through scholarships while studying, while some benefited by being appointed to transitional roles during their studies (NP4, NP7).

Once they had established a role, NPs were afforded some autonomy and, again, found ways to work around institutional barriers to deliver a high-quality service.

*… using the nursing model but being able to extend the scope of practice you’re able to provide a more comprehensive health service to that person and their family. … I am able to, and will manage within how I feel comfortable, looking at the renal disease and hypertension and infection and illnesses as well.* (NP1)

Meanwhile, secondary informants who had responsibility for overseeing services delivery, such as NP15C2, found value in national and state-based legislation that *‘standardised and clarified’* NP roles.

### Meso-barriers

At the meso-level were local institutional and community issues. In Australia, the NP role is reliant on State and National government funding, with local health services reluctant to commit funding to NPs, which are higher paid than RNs [44]. Informants questioned the genuine commitment of some managers.

*I’ve heard the Director of Nursing speak and he says he’s in favour of NPs … so if you’re in favour of nurse practitioners why did the funding for three positions sit on your desk for 12 months and go nowhere?* (NP3C).

Others noted stressors associated with limited staffing, inadequate breaks during shifts, fatigue and burnout. Those from remote, single-practitioner facilities fulfilled both clinical and administrative roles without assistance.

Some also identified a lack of recognition and understanding of their role in the community.

*So, I think that broader recognition from a national and societal perspective too. I don’t think the role is really understood by the community. There are a lot of questions from clients and patients about what do you do sort of thing. … it is just that broader societal understanding.* (NP2C)

When NP5 first started, he felt the need to explain his role to each patient but eventually stopped doing that. Other informants explained the difference between their role and that of the doctor (NP9, NP10, NP11), while another, NP8, became to be known as *'doc'* in one remote community. Some patients were surprised that NPs could write a prescription (NP7, NP12).

### Meso-enablers

Although the NP role was not widely known, NPs found that patients and carers were receptive once they understood the services the NP could provide.

*My community are very loyal to me. I’m booked out fully on my nurse practitioner days and patients have to wait a few weeks to get in, … I have* [Medicare] *provider numbers at both surgeries, so I actually don’t cost them a lot of money, …* (NP12)

NPs were reliant on formal and informal networks for professional support and advice. Some had established informal networks with other NPs or had built local networks of general practitioners (GPs) and medical specialists to call for advice, as well as advocacy.

*I spent a lot of time networking and trying to get good rapport with the GPs, particularly a lot of those that went to residential care facilities, and some GPs are fantastic and I’ll just ring them and like ‘yep, no dramas, whatever, not a problem … .* (NP7)

*When the senior medical officer for the district … is on-board with saying that the nurse practitioners should be doing it, it gets listened to by a lot of people.* (NP8)

In contrast to earlier examples of organisational resistance, there were instances where NPs found senior management supportive. Informants recounted circumstances where their local health service manager sought to implement the NP model, particularly for chronic disease management and primary health care. In some cases, NPs were supported with access to professional development, even access to rural generalist medical training and updates (NP8).

### Micro-barriers

The micro-level relates to day-to-day practice, in which NPs recounted stories of resistance from some other health professionals. This arose from poor understanding of the NP role, resulting in lack of support from colleagues. It often stemmed from issues of role clarity and relationships as new roles evolved.

*It's been an interesting journey … , so how do we work with the services that were pre-existing and that's taken two years to sort of work that out really. Lot of time and effort goes into sorting out the roles in relation to other pre-existing roles.* (NP14)

Some NPs did not have a clear job description when they commenced and it was left to them to define their own scope of practice.

*I had no job description, I had no idea what on earth my role was supposed to be. I just had to hit the ground running and start off by asking questions, seeing what was there and looking for gaps in places that I value, so I probably didn't see a patient for the first six months of my role* (NP11)*.*

In addition, in many instances, the presence of NPs clearly challenged traditional professional status and hierarchy. Some commented that disagreements resulted in interprofessional tension with doctors, for example: “*they remind you that you’re a nurse practitioner and not a medical specialist at that point, even when they’re wrong”* (NP2).

Working in isolation was a barrier for remote area NPs. For one NP working in a remote Aboriginal community it was difficult to organise annual leave coverage, there was little respite and, at times, exposure to personal risk.

*When you’re on-call, someone either rings your phone or bangs on your door in the middle of the night, … You don’t know whether it’s someone that’s drunk, someone that’s unwell, a child with meningitis or someone just wants a Panadol* [paracetamol]*.* (NP6).

### Micro-enablers

Not withstanding comments above under micro-barriers, resistance to the NP role from other health professionals was not universal. Some informants received support from other nurses, allied health professionals and doctors. Working in a team rather than as an isolated independent practitioner was an enabler to NP’s practice. One NP explained that she encountered significant resistance from nursing colleagues and would have resigned if not for the support from medical colleagues.

*I had a very supportive regional medical director, a cardiologist and very pro-nursing. … Also a GP that I work quite closely with, a female GP who does fly in-fly out work. Both of those two in terms of mentoring and support were fantastic. I would probably have quit if I didn't have the two of them* (NP11).

The capabilities of the NPs played an integral role in gaining acceptance from other health professionals and contributing to improvements in service delivery. This appeared to be an important micro-level enabler, with attributes of diplomacy, negotiation, resilience, advocacy and promotion of the role considered important.

*You have to get out there, you actually have to be the diplomat, be supportive of others and acknowledge their expertise as well and through that you will get buy-in into it. … be proactive and go on the front foot to gain the support of peers and other health professionals.* (NP1)

NPs explained that they had quickly learnt to tread carefully, acknowledging colleagues’ skills and expertise and building relationships.

*I had to say to people … 'You guys are doing a fantastic job and I'm not here to come in on my white charger solving every problem in the world because that's not I'm here for’.* (NP11)

For one NP, offering advice to GPs became less confrontational over time: *“I try to get them to tell me what their concerns are and then just gently tell them why they’re wrong”* (NP2). Another described how, through diplomacy and resilience, resistance eroded over time:

*All those places that were resistant have now asked me to consult, so I don't have any areas left now where people don't want me. … , just show what you can do, quietly get on with it, and eventually it will all sort itself out and that's what happened … you've got to be a real diplomat in this role* (NP11).

## Discussion

Drawing on established socio-institutional theory, this study has examined how health care professionals enact extended scope of practice roles in light of the combination of both formal (regulatory and institutional) and informal normative (interprofessional and interactional) factors that guide behaviour and shape practice [27–29]. Management and sociology literature [28], particularly relating to health professions [19], suggests that analysis of macro, organisational factors integrated with persistent informal constraints to lowering traditional barriers between professions at the meso- and micro-level can provide insights into barriers and enablers of innovative work practices. The study aimed to examine the ostensibly successful NP practice model to better understand the influences on the development of extended scope of practice more generally, making use of the macro, meso and micro socio-institutional structure.

In relation to the NP model, Haines and Critchley found a relatively narrow range of factors, categorised broadly into barriers and enablers [16]. Some, such as limited access to education, balanced against having some fee support and designated study time, were also apparent in this study at the macro-level (Fig. 1 and Table 2). Common barriers evident across several studies include: other health professionals negative perceptions or lack of awareness of the NP role [16, 18, 45] (micro-level); the inflexibility of the MBS and PBS funding model [16, 45, 46] (macro-level); workload issues, unclear career pathways and lack of peer or management support [41, 45, 46] (meso- and micro-level). Meanwhile, these were potentially balanced against enablers like: building support networks and local teamwork [16, 18, 45] (meso-level); and clarity of leadership and organisational structure [41, 45] (meso- and micro-level). Some studies broke down barriers and enablers into different levels similar to the structure used in this study, such as the healthcare system, organisational, team and individual practitioner levels [41] or the policy, workplace and personal levels [45]. For the most part, there is a considerable degree of cross-over between categories in this and previous studies, although the importance of community understanding and support was more apparent at the meso-level, as was the value placed on negotiation and advocacy of roles (micro-level) by informants in this study.

The concept of developing innovative models of care using vertical and horizontal ‘skill transfer’ is not new [47, 48]. Such initiatives have been considered and, in some cases implemented in other countries. Calls have also been made in Australia to boost the capacity of the rural health workforce by, for example, expanding the scope of pharmacists and facilitating the introduction of physician assistants [22, 23]. While some progress has been made in the renegotiation of role boundaries, it has been limited. Examining the evolution of NPs through an institutional lens may shed further light on the factors that inhibit the development of other extended scope of practice roles both in Australia and in other developed countries by providing a framework for other health professions to reflect on and potentially formalise extended and advanced practice roles.

Health service innovations are primarily governed by legislative and regulatory provisions and by policy at the macro- and meso-level. It is apparent from extant literature, as well as from the strong views of some informants in this study, that at the macro-level the MBS and PBS are barriers to innovative extended scope of practice models in Australia [16, 22, 43]. It has been argued elsewhere that consideration should be given to revising Medicare legislation and regulations that restrict access to the payment system for most health professionals and potentially increase the cost of health care. For example, the Grattan Institute estimated that a saving of $430 million per year could be made by extending the roles of health professionals [49]. While cost is not an isolated issue and service quality and patient safety are priorities, account must also be taken of risks to patients and practitioners of having to work-around perceived restrictive regulatory barriers in order to deliver optimal care in rural and remote locations where service access and availability are limited.

It was also perceived by respondents that barriers persisted around a lack of awareness and understanding of the NP role by managers, at the meso-level. Opportunities to implement NPs roles were either not realised, in which case positions went unfilled, or else funding was limited and short-term. There was a perception that health services were unwilling to commit funds to sustain NP roles, with funding directed elsewhere. Such findings reinforce perceptions that senior management lacks understanding of extended scope of practice roles and the potential to address health service gaps using innovative models of care [50, 51]. Established health service models and structures are often inflexible and not adaptable, with managers retaining allegiance to their clinical professional identity and having limited understanding of roles beyond their own occupational domain [52]. Consequently, NPs apparently spend considerable time explaining, negotiating and advocating for their role (micro-level); time that could perhaps be better spent providing patient care.

Research on health care human resources has identified the barrier created by ‘professional monopolies’ and a need to focus on the competencies necessary to perform tasks, rather than the protection of professional identities [53]. Recognition of specific competencies shared across professions requires disentangling competencies from professional repertoires and acknowledgement of individuals’ abilities to competently perform tasks to meet specific patient needs [53]. Indeed, the need to explain and advocate for their role and negotiate role boundaries, implies a need for high-level behavioural competencies and interactional skills in order to be effective in extended scope of practice roles generally, because of threats to professional jurisdiction, whether genuine or perceived.

At the mico-level, roles were most effective when NPs were supported by their nursing, medical and allied health colleagues with who they shared trust and understanding of each other’s roles. Such micro-level enablers seemed to counter-balance barriers associated with lack of role clarity at the boundary with medicine [54], as well as with other nurses and allied health professionals. Where such barriers exist, there is a risk of sabotage of extended roles due to professional jealousy or perceived threats to role distinctiveness [55].

Preservation of professional identity and hierarchical practice models are common features of the health care system, with the apparent dominance of the medical profession being institutionally embedded [56]. Medical resistance to the NP model has been identified previously [57, 58], arguably stemming from concerns about encroachment on medical scope of practice and threats to power and income [59, 60], which apparently manifest at the micro-level. Some NPs in this study were reminded of their status during professional disagreements with doctors, although details of such interactions were not explored from both sides. In other reported instances, NPs were more likely to find support from medical rather than nursing colleagues, validating perceptions that NPs are increasingly accepted by local doctors [54], particularly once the scope of practice and benefits are appreciated [61]. Additionally, once patients understood the scope of the role and the improved access to care, community support was also a strong meso-level enabler. Indeed, patient support for NP roles has been reported internationally [62, 63], largely linked to the longer consultations and the focus on patient education components of NP practice [64].

### Implications

Challenging the status quo of the health professional hierarchy and traditional models of practice is likely to manifest in predictable patterns, no matter which interprofessional boundary is crossed or shifted. Therefore, examination of barriers and enablers of the rural NP role has potential to inform the evolution other extended scope of practice roles in rural health, with the opportunity to proactively minimise future challenges. From this perspective there are some strong messages in this study. For example: clearly define and standardise the scope of practice, preferably within a regulatory framework (macro-level); ensure continuing educational and the development of support networks (meso- and macro-levels); appreciate the importance of negotiation with neighbouring occupational groups (micro-level); and promote the extended role to increase awareness of other health professionals and the community (meso- and micro-level). However, in Australia, the future development of extended scope of practice roles in other professions is undoubtedly restricted by the current funding model, a major barrier at the macro-level, as it has been for the NP role.

The findings of this study also have implications for human resource management in health care and the development of requisite competencies to maximise the effectiveness of extended scope and advanced practice roles. For those in leadership positions, staff shortages in rural and remote areas are serious challenges that can only be addressed by adequate planning at the meso-level. From a retention perspective, the findings echo those of previous studies [65], that leadership and supervisor–practitioner relationships are central to positive experiences, influencing intentions to stay, be it in the rural community generally [66] or specifically in an extended scope of practice role.

Health service managers and leaders, both within and beyond the immediate practice environment, must develop the necessary knowledge and abilities to advocate effectively for extended scope of practice roles. There is a palpable need for relations-oriented leadership behaviours aimed at building commitment and cooperation among different occupations [67, 68], both in specific workplaces and across the health care system. Managers and leaders can influence attitudes about and behaviours towards extended roles, as well as advocating across interprofessional, as well as intra- and inter-organisational boundaries. An important practical outcome of rural NP roles has been the devolution of care using, so called, ‘shared care’ models between NPs and local doctors [17]. The implications for health services is that the success of such innovative models of care is dependent on powerful ‘champions’ throughout the system [53], with the necessary relations-oriented leadership behaviours to moderate resistance to change.

### Strengths and weaknesses

Because in many parts of the world there is an enduring geographic maldistribution of health workforce, there is a persistent need to explore workforce planning and alternative models of care in rural communities [69]. This study makes a timely contribution in this context, extending the understanding of the barriers and enablers of extended scope of practice in health care. While some previous studies have provided insights into the practical challenges, this study used a multi-level, socio-institutional lens to examine the issues, a method that has its origins in business management. Few previous studies have examined the barriers and enablers to extended scope of practice roles from a multi-level perspective but none have so coherently integrated the practical insights with a socio-institutional lens.

The study sample of primary informants was small, with two having been NP candidates, not yet accredited. This reflects the need as well as the shortage of NPs in non-metropolitan locations, so they were included. Another limitation of the study is that the analysis was confined to NPs, suggesting the findings may be extrapolated to other disciplines. This approach may be questioned, given differences between the comparatively new, endorsed role of the NP and existing roles of other professions that may aspire to extend the scope of their practice. However, such an approach was necessitated by the lack of extended scope of practice roles in other disciplines in the Australian health care system, recognition of which motivated this study. A further opportunity exists to re-examine the application of the multi-level, socio-institutional lens with health professions in countries other than Australia where extended scope of practice roles are more common and diverse.

## Conclusion

NPs provide valuable services in rural and remote communities. The nurse practitioner model illustrates many of the enablers and barriers to the development of extended scope of practice in other health professions and is a valuable source of several lessons. Two key service and policy recommendations arise from this study. Firstly, if underserved communities are to benefit from innovative models of health service delivery, including extended scope of practice, energy needs to be directed towards addressing legislative and regulatory barriers, such as the MBS and PBS in this case study. Secondly, there needs to be a focus on promoting extended scope of practice roles with policy and decision-makers in health care, from the perspective that long-term efficiency gains and cost-savings can be achieved only if short-term investment in innovation is supported and sustained by policy initiatives.

## Additional file

Additional file 1: Schedule of questions used for primary and secondary informant interviews. Lists of questions used in qualitative interviews with rural nurse practitioners in Australia. (DOCX 29 kb)

## Abbreviations

GP: General practitioner
MBS: Medicare Benefits Schedule
MMM: Modified Monash Model
NP: Nurse practitioner
NSW: New South Wales
PBS: Pharmaceutical Benefits Scheme
RN: Registered nurse

## References

[1] Health Workforce Australia. Australia’s Future Health Workforce - Nurses Detailed. 2014. http://www.health.gov.au/internet/main/publishing.nsf/content/australias-future-health-workforce-nurses. Accessed 22 Mar 2019.

[2] Health Workforce Australia. Australia’s Future Health Workforce – Doctors. 2014. http://www.health.gov.au/internet/main/publishing.nsf/Content/australias-future-health-workforce-doctors. Accessed 22 Mar 2019.

[3] World Health Organisation (WHO). Increasing access to health workers in remote and rural areas through improved retention. 2010. http://www.searo.who.int/nepal/mediacentre/2010_increasing_access_to_health_workers_in_remote_and_rural_areas.pdf. Accessed 22 Mar 2019. 23741785

[4] Australian Government. National Strategic Framework for Rural and Remote Health. 2012. http://www.health.gov.au/internet/main/publishing.nsf/content/national-strategic-framework-rural-remote-health. Accessed 22 Mar 2019.

[5] Australian Government Productivity Commission. Australia's Health Workforce Research Report. 2005. http://www.pc.gov.au/inquiries/completed/health-workforce/report/healthworkforce.pdf. Accessed 22 Mar 2019.

[6] Health Workforce Australia (HWA). National Rural and Remote Workforce Innovation and Reform Strategy. 2013. http://pandora.nla.gov.au/pan/133228/20150419-0017/www.hwa.gov.au/sites/uploads/HWA13WIR013_Rural-and-Remote-Workforce-Innovation-and-Reform-Strategy_v4-1.pdf. Accessed 22 Mar 2019.

[7] World Health Organization. Task Shifting: Rational Redistribution of Tasks Among Health Workforce Teams: Global Recommendations and Guidelines. 2007. http://apps.who.int/iris/handle/10665/43821. Accessed 22 Mar 2019.

[8] Fairman JA, Rowe JW, Hassmiller S, Shalala DE (2011). Broadening the scope of nursing practice. N Engl J Med.

[9] Harvey C (2011). Legislative hegemony and nurse practitioner practice in rural and remote Australia. Health Sociol Rev.

[10] Mills J, Lindsay D, Gardner A (2011). Nurse practitioners for rural and remote Australia: creating opportunities for better health in the bush. Aust J Rural Health.

[11] Bauer JC (2010). Nurse practitioners as an underutilized resource for health reform: evidence-based demonstrations of cost-effectiveness. J Am Acad Nurse Pract.

[12] Nursing and Midwifery Board of Australia. Nurse and Midwife – Registration Table – December 2016. http://www.nursingmidwiferyboard.gov.au/About/Statistics.aspx. Accessed 22 Mar 2019.

[13] Scanlon A, Cashin A, Bryce J, Kelly JG, Buckely T (2016). The complexities of defining nurse practitioner scope of practice in the Australian context. Collegian..

[14] Duffield C, Gardner G, Chang AM, Catling-Paull C (2009). Advanced nursing practice: a gobal perspective. Collegian.

[15] Gardner G, Duffield C, Doubrovsky A, Adams M (2016). Identifying advanced practice: a national survey of a nursing workforce. Int J Nurs Stud.

[16] Haines HM, Critchley J (2009). Developing the nurse practitioner role in a rural Australian hospital – a Delphi study of practice opportunities, barriers and enablers. Aust J Adv Nurs.

[17] Roots A, MacDonald M (2014). Outcomes associated with nurse practitioners in collaborative practice with general practitioners in rural settings in Canada: a mixed methods study. Hum Resour Health.

[18] Leidel S (2014). Nurse practitioners in Australia: strategic errors and missed opportunities. Med J Aust.

[19] Currie G, Lockett A, Finn R, Martin GP, Waring J (2012). (2012). Institutional work to maintain professional power: recreating the model of medical professionalism. Organ Stud.

[20] Productivity Commission. Australia’s health workforce, research report, Canberra. https://www.pc.gov.au/inquiries/completed/health-workforce/report. Accessed 22 Mar 2019.

[21] Mason J. Review of Australian Government Health Workforce Programs. Canberra, Commonwealth of Australia. 2013. Available: https://www.health.gov.au/internet/main/publishing.nsf/Content/D26858F4B68834EACA257BF0001A8DDC/$File/Review%20of%20Health%20Workforce%20programs.pdf. Accessed 22 Mar 2019.

[22] Productivity Commission 2015, Efficiency in health, commission research paper, Canberra. JEL codes: I10, I18. https://www.pc.gov.au/research/completed/efficiency-health/efficiency-health.pdf. Accessed 22 Mar 2019.

[23] Duckett S, Breadon P, Ginnivan L. Access all areas: new solutions for GP shortages in rural Australia. Grattan Institute, Melbourne. 2013. https://grattan.edu.au/report/access-all-areas-new-solutions-for-gp-shortages-in-rural-australia/. Accessed 22 Mar 2019.

[24] Maier CB, Barnes H, Aiken LH, Busse R. Descriptive, cross-country analysis of the nurse practitioner workforce in six countries: size, growth, physician substitution potential. BMJ open. 2016*;6*(9):e011901. 2016. 10.1136/bmjopen-2016-011901. 10.1136/bmjopen-2016-011901 PMC5020757 27601498

[25] Niezen MGH, Mathijssen JJP (2014). Reframing professional boundaries in healthcare: a systematic review of facilitators and barriers to task reallocation from the domain of medicine to the nursing domain. Health Policy.

[26] Allied Health Professions Office of Queensland, Queensland Health. Ministerial taskforce on health practitioner expanded scope of practice: Final report. June 2014. https://www.health.qld.gov.au/__data/assets/pdf_file/0031/161977/ministerial-taskforce-report.pdf. Accessed 22 Mar 2019.

[27] Scott WR. Institutions and organizations. London: Sage. 1995.

[28] Scott WR (2008). Lords of the dance: professionals as institutional agents. Organ Stud.

[29] Hodgson GM (2006). What are institutions?. J Econ Issues.

[30] Simons H. Case study research in practice: Sage publications; 2009.

[31] Punch KF. Introduction to social research: quantitative and qualitative approaches: Sage; 2013.

[32] Crossley M, Arthur L, McNess E (2016). Revisiting insider-outsider research in comparative and international education.

[33] Huberman AM, Miles MB, Denzin NK, Lincoln YS. (1994). Handbook of qualitative research. Data management and analysis methods. Thousand oaks, CA, sage: 2010.

[34] Andersen PH, Kragh H (2010). Sense and sensibility: two approaches for using existing theory in theory-building qualitative research. Ind Mark Manag.

[35] Joffe H, Yardley L, Marks D, Yardley L (2004). Content and thematic analysis. Research methods for clinical and Health Psychology.

[36] Corbin JM, Strauss AL (2015). Basics of qualitative research: Techniques and procedures for developing grounded theory. Thousand Oaks.

[37] Glaser BG (1978). Theoretical Sensitivity: Advances in the Methodology of Grounded Theory.

[38] Australian Government Department of Health. Doctor Connect: Rural Classification Reform, Modified Monash Model – Frequently Asked Questions. [Cited 28 June 2016] Available from: http://www.doctorconnect.gov.au/internet/otd/publishing.nsf/content/classification-changes. Accessed 22 Mar 2019.

[39] Mulvale G, Embret, M, Razavi SD. 'Gearing Up' to improve interprofessional collaboration in primary care: a systematic review and conceptual framework. BMC Family Practice. 2016;17(1):83. doi: 10.1186/s12875-016-0492-1 10.1186/s12875-016-0492-1 PMC4955241 27440181

[40] Nelson S, Turnbull J, Bainbridge L, Caulfield T, Hudon G, Kendel D, et al. Optimizing Scopes of Practice: New Models for a New Health Care System. Ottawa, Ontario. http://www.cahs-acss.ca/wp-content/uploads/2014/08/Optimizing-Scopes-of-Practice_REPORT-English.pdf. Accessed 22 Mar 2019.

[41] Elliott N, Begley C, Sheat G, Higgins A. Barriers and enablers to advnaced practitioners’ ability to enact their leadership role: a scoping review. Int Journal Nurs Stud. 2016; 10.1016/j.inurstu.2016.03.001. 10.1016/j.ijnurstu.2016.03.001 27297366

[42] Cashin A (2014). Collaborative arrangements for Australian nurse practitioners: a policy analysis. J Am Acad Nurse Pract.

[43] Cashin A, Theophilos T, Green R (2016). The internationally present perpetual policy themes inhibiting development of the nurse practitioner role in the primary care context: an Australian–USA comparison. Collegian..

[44] Lowe G, Plummer V, Boyd L (2013). Nurse practitioner roles in Australian healthcare settings. Nurs Manag.

[45] Francis K, Anderson J, Mills N, Hobbs T, Fitzgerald M (2013). Advanced roles for nurse working in general practice: a study of barriers and enablers for nurses in rural Australia. Clin Nurs Stud.

[46] McKenna L, Halcomb E, Lane R, Zwar N, Russell G (2015). An investigation of barriers and enablers to advanced nursing roles in Australian general practice. Collegian.

[47] Brooks PM, Lapsley HM, Butt DB (2003). Medical workforce issues in Australia: tomorrow’s doctors – too few, too far. Med J Aust.

[48] Duckett SJ (2005). Health workforce design for the 21st century. Aust Health Rev.

[49] Duckett and Breadon, P. Unlocking Skills in Hospitals: Better Jobs, More Care, Grattan Institute, Melbourne; 2014.

[50] Keating SF, Thompson JP, Lee GA (2010). Perceived barriers to the sustainability and progression of nurse practitioners. Int Emerg Nurs.

[51] Middleton S, Gardner A, Della PR, Lam L, Allnutt N, Gardner G (2016). How has the profile of Australian nurse practitioners changed over time?. Collegian..

[52] Briggs D, Cruickshank M, Paliadelis P (2012). Health managers and health reform. J Manag Organ.

[53] Nancarrow SA (2015). Six principles to enhance health workforce flexibility. Hum Resour Health.

[54] MacLellan L, Higgins I, Levett-Jones T (2015). Medical acceptance of the nurse practitioner role in Australia: a decade on. J Am Acad Nurse Pract.

[55] MacLellan L, Higgins I, Levett-Jones T (2016). An exploration of the factors that influence nurse practitioner transition in Australia: a story of turmoil, tenacity, and triumph. J Am Assoc Nurse Pract.

[56] McNeil K, Mitchell R, Parker V (2013). Interprofessional practice and professional identity threat. Health Sociol Rev.

[57] Elsom S, Happell B, Manias E (2009). Nurse practitioners and medical practice: opposing forces or complementary contributions?. Perspect Psychiatr Care.

[58] McMurray R (2011). The struggle to professionalize: an ethnographic account of the occupational position of advanced nurse practitioners. Hum Relat.

[59] Appel AL, Malcolm P (2002). The triumph and continuing struggle of nurse practitioners in New South Wales. Australia Clin Nurse Spec.

[60] Turner C, Keyzer D, Rudge T (2007). Spheres of influence or autonomy? A discourse analysis of the introduction of nurse practitioners in rural and remote Australia. J Adv Nurs.

[61] Lloyd-Rees J (2016). How emergency nurse practitioners view their role within the emergency department: a qualitative study. Int Emerg Nurs.

[62] Jennings N, Clifford S, Fox AR, O'Connell J, Gardner G (2015). The impact of nurse practitioner services on cost, quality of care, satisfaction and waiting times in the emergency department: a systematic review. Int J Nurs Stud.

[63] McDonnell A, Goodwin E, Kennedy F, Hawley K, Gerrish K, Smith C (2015). An evaluation of the implementation of advanced nurse practitioner (ANP) roles in an acute hospital setting. J Adv Nurs.

[64] Clark S, Parker R, Prosser B, Davey R (2013). Aged care nurse practitioners in Australia: evidence for the development of their role. Aust Health Rev.

[65] Brunetto Y, Shriberg A, Farr-Wharton R, Shacklock K, Newman S, Dienger J (2013). The importance of supervisor–nurse relationships, teamwork, wellbeing, affective commitment and retention of north American nurses. J Nurs Manag.

[66] Schoo AM, Stagnetti KE, Mercer C, Dunbar J. A conceptual model for recruitment and retention: Allied health workforce enhancement in Western Victoria, Australia. Rural Remote Health 2005;5:477 (online). 16375575

[67] Gilbert C, De Winne S, Sels L (2011). The influence of line managers and HR department on employees' affective commitment. Int J Hum Resour Man.

[68] Yukl G (2008). How leaders influence organizational effectiveness. Leadersh Q.

[69] MacLeod ML, Stewart NJ, Kulig JC, Anguish P, Andrews M, Banner D (2017). Nurses who work in rural and remote communities in Canada: a national survey. Hum Resour Health.

