# Supplementary material for: A study of macro-, meso- and micro-barriers and enablers affecting extended scopes of practice: the case of rural nurse practitioners in Australia
Source: BMC Nurs. 2019 Apr 2;18:14. doi: 10.1186/s12912-019-0337-z (PMC6444450; doi:10.1186/s12912-019-0337-z)
Supplement: Supplementary file 1 — Schedule of questions used for primary and secondary informant interviews. Lists of questions used in qualitative interviews with rural nurse practitioners in Australia. (DOCX 29 kb) [file 12912_2019_337_MOESM1_ESM.docx]

**Additional file**

| **Primary Informants’ Questions** |
| --- |
| - Please describe the extended scope of practice role that you undertake? |
| - How do you perceive this role extends outside the traditional role of your health profession? |
| - What motivated you to take on the extended scope of practice role? |
| - What factors help you to take on the extended scope of practice role? |
| - How, if at all, have other staff and colleagues responded to your extended role? |
| - How, if at all, do patients or their carers respond to your extended role? |
| - What, if any, have been the positive outcomes of your extended scope of practice role? |
| - What, if any, have been the negative outcomes of your extended role? |
| - What things, if anything, worry you or make you feel less secure in your extended role? |
| - Is the extended role what you expected it to be?   - Are there things that you have found out about performing in your extended role that you had not expected? |
| - Are there any other things that have helped you to adopt an extended scope of practice?   - What things have made it difficult? |
| - Are there any other things about performing your extended role that you can comment on? |
| **Secondary Informants’ (Colleagues’) Questions** |
| - Please describe your relationship with [the primary participant] and your role. |
| - Please describe the extended scope of practice role of [the primary participant]. |
| - In what ways do you perceive that role extends outside the traditional role boundaries of their health profession? |
| - How, if at all, have other staff members and colleagues reacted since [the primary participant] took on the role?   - Has their behaviour and attitude changed? |
| - What do you think are the main outcomes of the extended role? |
| - What have been the positive outcomes of your colleague’s extended role? |
| - What are the things, if anything, which might worry you about their extended role? |
| - Are there any things that helped your colleague adopt an extended role   - What things have made it difficult? |
| - Are there any other things about the extended role that you want to comment on? |

Schedule of questions used for primary and secondary informant interviews.
